# Breaking beta: deconstructing the parasite transmission function

**DOI:** 10.1098/rstb.2016.0084

**Published:** 2017-03-13

**Authors:** Hamish McCallum, Andy Fenton, Peter J. Hudson, Brian Lee, Beth Levick, Rachel Norman, Sarah E. Perkins, Mark Viney, Anthony J. Wilson, Joanne Lello

**Affiliations:** 1 Environmental Futures Research Institute, Griffith University, Nathan 4111, Queensland, Australia; 2 Institute of Integrative Biology, University of Liverpool, Liverpool L69 7ZB, UK; 3 Center for Infectious Disease Dynamics, Penn State University, University Park, PA 16802, USA; 4 School of Natural Sciences, University of Stirling, Stirling FK9 4LA, UK; 5 School of Biosciences, Cardiff University, Museum Avenue, Cardiff CF10 3AX, UK; 6 Department of Biodiversity and Molecular Ecology, Research and Innovation Centre, Fondazione Edmund Mach, Via E. Mach 1, 38010 S. Michele all'Adige, Trentino, Italy; 7 School of Biological Sciences, University of Bristol, Tyndall Avenue, Bristol BS8 1TQ, UK; 8 Vector-borne Viral Diseases Programme, The Pirbright Institute, Ash Road, Pirbright, Woking GU24 0NF, UK

**Keywords:** infection, infectious disease, modelling, nonlinearities, heterogeneity, transmission function

## Abstract

Transmission is a fundamental step in the life cycle of every parasite but it is also one of the most challenging processes to model and quantify. In most host–parasite models, the transmission process is encapsulated by a single parameter *β*. Many different biological processes and interactions, acting on both hosts and infectious organisms, are subsumed in this single term. There are, however, at least two undesirable consequences of this high level of abstraction. First, nonlinearities and heterogeneities that can be critical to the dynamic behaviour of infections are poorly represented; second, estimating the transmission coefficient *β* from field data is often very difficult. In this paper, we present a conceptual model, which breaks the transmission process into its component parts. This deconstruction enables us to identify circumstances that generate nonlinearities in transmission, with potential implications for emergent transmission behaviour at individual and population scales. Such behaviour cannot be explained by the traditional linear transmission frameworks. The deconstruction also provides a clearer link to the empirical estimation of key components of transmission and enables the construction of flexible models that produce a unified understanding of the spread of both micro- and macro-parasite infectious disease agents.

This article is part of the themed issue ‘Opening the black box: re-examining the ecology and evolution of parasite transmission’.

## 1. Introduction

Parasitism is a particular form of consumer–resource interaction [1] in which a consumer individual (the parasite; hereafter referring to both macroparasites and microparasites) lives on or within one resource individual (the host). Transmission of parasites between individual hosts is thus critical to the fitness of the parasite. Nonlinearities in transmission may have profound effects on the population dynamics of the parasite [2], and by extension the host. Despite the potential complexities in the biology of transmission, the vast majority of host–parasite models encapsulate the process in a single parameter, conventionally represented as *β*, the ‘transmission coefficient’. This single parameter encompasses two fundamental processes, which themselves comprise multiple sub-processes: (i) the contact rate between susceptible (*S*) and infectious (*I*) individuals; and (ii) the subsequent likelihood that transmission will occur during a contact [3–5]. Much of the discussion regarding the validity of the *β* transmission term has concentrated on the contact rate element of *β*, producing a variety of functional forms of the contact rate between infectious and susceptible individuals. In particular, at the population level, transmission of micro-parasites is often dichotomized into one of two simplified forms that are based on assumptions about how transmission-relevant contacts scale with population size [4]: density-dependent transmission, where transmission is assumed to occur at a rate *βSI*, or frequency-dependent transmission, where transmission is at a rate *βSI/N* (where *N* is total host population size) [6]. Broadly speaking, frequency-dependent transmission is typically assumed to be most appropriate for vector-borne and sexual transmission, where the number of contacts is assumed to be constant regardless of population size, whereas density-dependent transmission is typically the default assumption for most other modes of transmission [6]. For direct life cycle macroparasites, *β* often represents the contact rate between transmission stages and hosts [7]. Although convenient from a theoretical perspective, this discrete classification of transmission modes has often been shown to be incorrect when challenged with empirical data: some sexually transmitted infections have been shown to be more-closely represented by a density-dependent transmission function [8], whereas some directly transmitted infections have been shown to be better represented by frequency-dependent transmission functions [9]. Indeed, when alternative transmission functions have been applied to empirical data, they have often been shown to be preferable to either of these standard formulations, implying that the ‘real’ transmission process either lies on a continuum between these two extreme forms (e.g. ∼*βSI/N^q^*, where 0 < *q* < 1) [10], or that it is more complex than can be captured by any combination of these forms [11–13]. Scale can also be an issue as all transmission events can be considered as frequency-dependent when we consider just the contact of individuals and yet the closest approximation when we scale to population size could be density-dependent transmission. For example, density-dependent transmission captures the dynamics of measles at the level of a city and yet at the scale of the classroom frequency-dependent transmission would probably work better [14].

Clearly, the simplified forms of the transmission function described above are mathematically convenient abstractions of a sequence of distinct, but interacting, stages, through which an infection is transmitted from one individual to another [5]. These stages may each contain substantial nonlinearities, potentially differing for parasites with different transmission modes, that will alter the functional form of the overall transmission process and so the suitability of the standard mathematical transmission functions. Here, we unpick these processes, outlining a single transmission framework that applies for all forms of parasite transmission. We then show how this overall framework can be used to describe different parasites with different transmission modes, by considering the sequence of the various steps along the transmission pathway. Next, we describe how nonlinearities can emerge at each of the steps of this pathway, before exploring mathematically how these nonlinearities alter the shape of the overall transmission process, and drive departures from the ‘standard’ transmission function. Finally, we consider if and when the current practice of encapsulating transmission in a single parameter *β* is legitimate, and under what circumstances a more complex modelling framework may be required.

## 2. Deconstructing *β*

**Figure 1.**
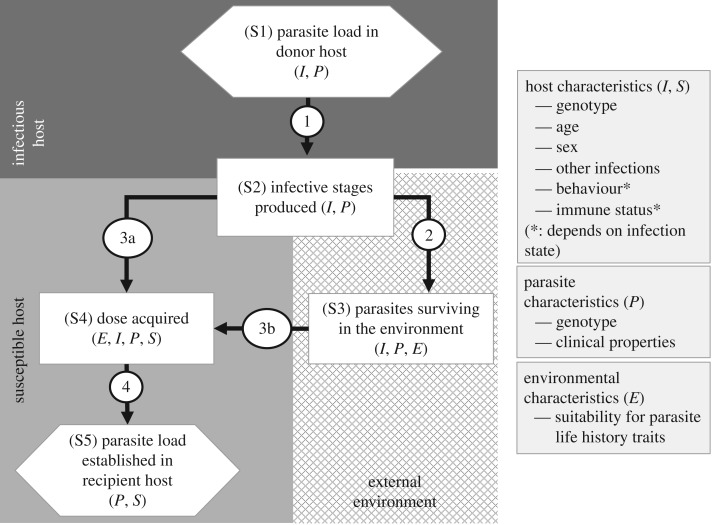
Schematic decomposition of the transmission process. Hexagons represent parasite load in the donor (S1) and recipient (S5) hosts. Squares represent distinct stages of the transmission process and arrows represent transmission between stages. The letters *I* (infected host), *P* (parasite), *E* (environment) and *S* (susceptible host) represent potentially important factors acting at each stage relating to infectious (donor) host (*I*), the parasite, the environment and the susceptible (recipient) host (*S*), respectively.

Figure 1 shows a generalized transmission process, which is applicable to a single transmission event for a single genotype (or in some cases phenotype) of any parasite, whether a macroparasite or microparasite. This framework describes the relationship between parasite load in a single infected donor host individual (figure 1; hexagon ‘S1’) and the resulting established parasite load in a single recipient susceptible host (figure 1; hexagon ‘S5’). The relationship between donor and recipient parasite load is determined by a series of stages within the transmission process, which represent the abundance of parasites at each stage (rectangles in figure 1), linked by a series of transitions which describe the processes that alter those abundances (arrows in figure 1). The different stages, for different parasite types, may be influenced to varying extents by a range of intrinsic (i.e. parasite related) and extrinsic (i.e. donor host, recipient host, other parasites, wider environmental) factors leading to heterogeneity in the parasite load at each intermediary stage in the transmission process. Further, the functional form of the various transitions may be nonlinearly related to the parasite load in the preceding stage, and so we mathematically investigate the potential effects of these nonlinear relationships below.

Stage 1 of our framework (S1) represents the parasite load (of a given genotype or phenotype) within the donor-infectious host. Transition 1 denotes the functional relationship between this parasite load and the production of parasite infective stages (stage 2). These infective stages may or may not enter the external environment, and we therefore describe two qualitatively different pathways from this point. In one pathway, transmission occurs by direct contact between a susceptible recipient and infected donor host without infective stages entering the external environment at any point (figure 1, stages 1, 2, 4 and 5 and transitions 1 → 3a → 4). An example of this is trophic transmission, in which a susceptible host consumes part or all of a donor-infectious host, thereby acquiring infective stages. This pathway also includes vector transmission, infection via blood- or sap-feeding, and some vertical transmission such as trans-placental infection. The second pathway involves infective stages entering, if only briefly (see below), the external environment (figure 1, stages 1 through 5, and transitions 1 → 2 → 3b → 4); for parasites following this second pathway, transition 2 describes the relationship between the number of parasites produced and the number of parasites that survive in the environment (stage 3).

Transitions 3a and 3b describe the relationship between the number of infective stages produced (stage 2), or those that enter and survive in the environment (stage 3), and the number contacting the recipient host as a potentially infectious contact. Stage 4 therefore captures the exposure dose for the recipient host, which can be thought of as those infectious stages physically in contact with the host, but not yet established in or on that host. Transition 4 describes the functional relationship between the dose acquired by the recipient host and the outcome of infection, in terms of the newly established parasite load in the recipient host (the received parasite load; stage 5). Transition 4 therefore differentiates between the dose of parasites that initially contact, and the dose that actually establishes in the hosts. The overall relationship between the received parasite load in the recipient host at stage 5, and the parasite load in the donor host at stage 1 describes the overall transmission function for the parasite. Note that this framework describes the steps involved for a single transmission process (i.e. the link between an established infection in one host and a new infection in another host). For vectored parasites and those with transmission via an intermediate host, it is necessary to pass through our framework twice, once for each host transmission event.

## 3. Sources of heterogeneity and nonlinearity

Here, we describe potential sources of nonlinearities for each transition in figure 1, and their potential consequences for each of the subsequent stages, given a defined, starting parasite load in the donor host (stage 1). We then discuss the heterogeneities that can occur in each of the remaining stages, 2 through 5, some of which may themselves give rise to nonlinear effects in the transmission process or produce variance that is non-normally distributed. It is important to note that the parasite load at stage 1 is influenced by a wide range of within-host processes [5], a full exploration of which are beyond the scope of a discourse on transmission.

### (a) Reaching stage 2: number of infective stages produced

For many parasites, the relationship between the infection intensity in the donor host and its production of infective stages may be linear (e.g. amount of *Escherichia coli* shed in faeces [15]) but nonlinearities can also arise. For example, for many helminths it is well known that egg production is nonlinearly related to parasite intensity, due to density-dependent processes acting within individual hosts; either due to physical crowding of parasites or through increased activation of the host's immune response [16–18]. Density-dependent processes will tend to produce saturating (or even non-monotonic) relationships between increasing parasite load in a donor host, and the production of infective stages. Conversely, for enteric pathogens such as cholera, high infectious burdens can trigger diarrhoea or vomiting, releasing large numbers of infectious stages, while mild infections may not trigger the same severity of symptoms and hence result in lower parasite production. At a population level, or at the individual level through time, heterogeneities can arise at stage 2. The production of infective stages can often be highly variable through time from the same individual donor host because of host experience of infection or temporal variation in parasite reproduction (e.g. cercarial production in snails [19]). Further, two hosts could have similar levels of parasite intensity but infective stage production might vary greatly if, for example, one host has been recently infected while another is in the process of attacking and ejecting worms [20]. Nested models, scaling from within-host to between-host processes, have sought to address some of these heterogeneities in transmission dynamics by, for example, allowing the age of infection and parasite loads [21,22] to affect the transmission, but such models only deal with transition 1 of our defined transmission process. Direct competition among different parasites and/or cross-immunity among different parasites, may alter the production of infective stages by the focal parasite. For example, in HIV, the amount of virus produced may depend on the presence of ulcers or lesions and even the presence of gastro-intestinal worms [23]. Taken together, the characteristics of the focal parasite, the infectious donor host and any co-infecting parasites may all interact with one another to influence the production and output of the infectious stages in stage 2.

### (b) Reaching stage 3: parasite survival and dispersal in the external environment

Once infective stages have been produced they may or may not enter into the external environment (transitions 2 or 3a, respectively). For those entering the external environment, those free-living stages may compete for resources in the environment, potentially producing nonlinear, density-dependent survival/dispersal [24]. For example, infective stages of some parasites are important food sources for predators [25], potentially generating the entire suite of nonlinear predator–prey dynamics in the infective stage population. If parasitic infective stages enter the external environment (even briefly, such as measles virus sneezed into the air), then heterogeneities can arise due to: (i) the parasite, for example its response to particular environmental conditions [26]; (ii) the infected host, for example if differences in behaviour influence where infectious stages are deposited or dispersed [27,28]; and (iii) the external environment including the (micro)climate and physical structure [29].

### (c) Reaching stage 4: dose acquired

Nonlinearity can occur here when the number of produced parasite infective stages (stage 2) or the parasites surviving in the environment (stage 3) affect the chances of one another contacting the host. There are likely to be many sources of nonlinearity acting at transitions 3a and 3b, involving complex relationships between the density of recipient and donor hosts (transition 3a) or between recipient hosts and infective parasite stages (transition 3b), and this has perhaps been the greatest focus of research into nonlinearities in transmission [6,10,12,30]. These nonlinearities mean standard formulations of transmission may be inadequate. Density-dependent transmission, as represented by *βSI*, assumes susceptible hosts and infectious stages are fully mixed and move randomly with respect to each other. This is not the case in most host–parasite systems. Infective stages may actively search out susceptible hosts [31], or susceptible hosts may avoid areas contaminated with infective stages [32], all of which could introduce non-random patterns of mixing between parasite infective stages and hosts. Similarly, for sexually transmitted parasites, the contact rate is determined by mating behaviour, often generating high levels of heterogeneity and potential nonlinearities in contact rates [33]. For trophically transmitted parasites, the rate of prey consumption per predator is described by the predator's ‘functional response’ (the relationship between prey density and ingestion rate), as is the attack rate of a vector [30]. These functional responses are conventionally categorized as type I, type II and type III, with type I being linear (predator ingestion rate continuously increases with prey density), and types II and III being nonlinear, saturating at high prey densities due to constraints on the number of prey that can be handled per unit time. While type II smoothly approaches the asymptote, type III functional responses show additional nonlinearities, increasing sigmoidally to saturation, often attributed to predators switching among alternative prey types. Antonovics *et al.* [30] show that a type II functional response generates frequency-dependent transmission if the handling time (time required for the predator to handle a single prey item) is large, and density-dependent transmission if the handling time is short. This suggests not only that ‘true’ transmission functions are likely to lie between the density- and frequency-dependent extremes, but are likely to slide between them, depending on density. Furthermore, as a type III functional response is often generated by predators switching to a new prey species, generalist vectors may exhibit a type III functional response, switching from one host species to another depending on the relative abundance of the different species. These predator–prey feeding relationships will clearly introduce population level nonlinearities into the transmission dynamics of the associated trophically transmitted parasites (e.g. malaria). In addition, these relationships may be further modified if the parasite manipulates the intermediate host (e.g. through behavioural or morphological changes) to make it more prone to predation by the definitive host predator [34].

### (d) Stage 5: parasite load established in recipient host

There is a large literature on dose–response relationships for a range of parasites [35–37], which may produce nonlinearities that act on transition 4. Many studies presume the existence of a threshold dose, below which infection does not occur or is unlikely, though empirical evidence for such thresholds is limited and is an important area for future research. Heterogeneities influencing this final stage include (i) parasite attributes (i.e. quality of the infectious particles), influenced in turn by parasite phenotype/genotype (e.g. virulence factors, immunosuppressive capabilities), (ii) recipient host attributes (e.g. genetics, immune responsiveness), (iii) other parasites (e.g. host immune biasing, competitive exclusion in a site of infection) and (iv) route of infection (e.g. the infectious dose for a pulmonary versus cutaneous or gastro-intestinal infection can be different and result in variation in values of *β*). Parasite traits may also induce nonlinear effects: for example, quorum sensing among bacteria and yeast, which involves signalling between bacteria (or yeast cells) that requires threshold levels of autoinducer chemicals in interaction with bacterial density, in order for signalling to occur [38]. Such nonlinear quorum sensing can lead to biofilms which increase the probability of bacteria establishing infection [39]. Heterogeneities may occur in this state due to the within-host environment of the recipient. For example, establishment of larvae of the nematode *Ostertagia circumcincta* in cattle hosts is affected by abomasal pH, which in turn can be affected by factors such as the animals’ food [40] or other parasites [41]. Similarly, attributes of the host can affect establishment, for example, a host with a prior history of the parasite might have developed some resistance. Physiological traits may also play a role here, for example, hosts with sickle cell anaemia are more resistant to red blood cell invasion by malaria [42].

## 4. Implications of nonlinearities for the overall transmission function

**Figure 2.**
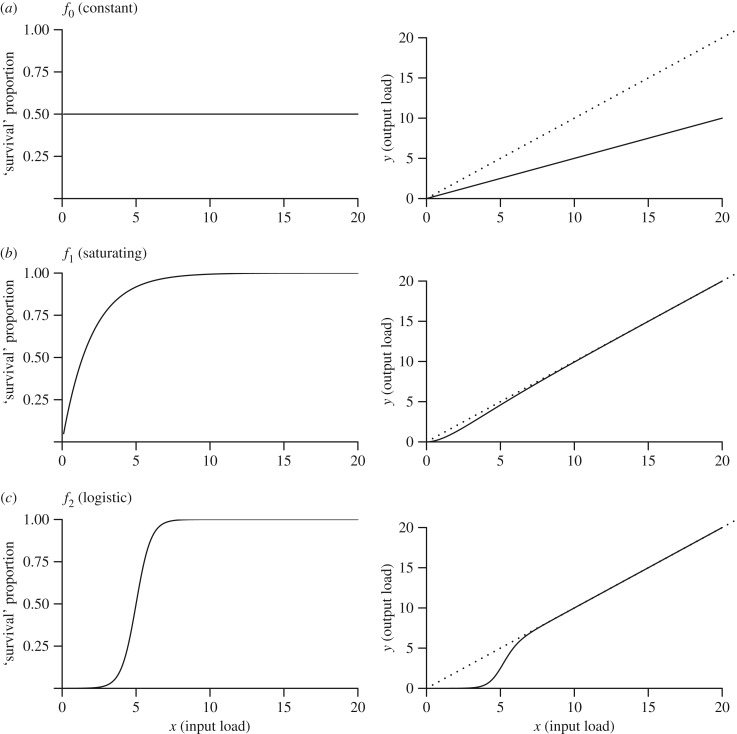
Graphical representation of the functional forms used in the transmission stage, where the *x*-axis is the ‘input’ parasite load into each function (i.e. the load from the previous step in the pathway) for: (*a*) linear function, (*b*) saturating function and (*c*) logistic function. Left-hand column: the *y*-axis shows parasite ‘survival’ (proportion of parasite load completing that stage); right-hand column: absolute ‘output’ parasite load (*y*) completing that stage. The dotted lines show the 1 : 1 relationship. Parameter *b* = 0.5, *c* = 0.5, *g* = 2 and *x*_0_ = 5.

Here, we explore mathematically how nonlinearities at the various points of the deconstructed transmission process influence the relationship between the parasite load within a donor host (*L*), and the resultant newly established load within the recipient host (*P*). For simplicity, we focus on the direct contact route (left-hand, 3a, route, figure 1), although the framework can easily be extended to incorporate additional nonlinearities during an explicit environmental stage (right-hand, 3b, route, figure 1). The function linking the recipient host's newly established parasite load (*P*) to the donor host's parasite load (*L*) is then a composite of three functions: *r*(*L*), the quantity of parasite infection stages encountered by the recipient as a function of the donor host–parasite load *L*; *d*(*r*), the parasite dose acquired by the recipient host as a function of the parasite infection stages encountered; and *p*(*d*), the resultant parasite load established in the recipient host as a function of the dose acquired by the recipient host. Thus, the resultant parasite load in the newly infected individual (*P*) is
4.1
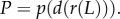
Each of these functions has the general form
4.2
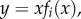
where we assume that the *f_i_*(*x*) are ‘survival’ functions (see below for specific functions considered, all with *f_i_*(*x*) < 1), which link the proportion of parasite load going into the next stage (y) with the parasite load coming into the current stage (x). We note that the initial step representing the production of infective stages as a function of parasite load in the donor host, *r*(*L*), could exceed 1 (e.g. for helminths where *per capita* fecundity is more than 1). We do not deal with this scenario explicitly here; to do so would require more in-depth consideration of both the functional form and magnitude of the relationship between parasite load (e.g. worm burden) and *per capita* fecundity. Such considerations are beyond the intended scope of the illustrative case we present here, so we restrict our assessment to ‘survival’ functions (*f_i_*(*x*) < 1). To capture a range of plausible relationships, we allow these *f_i_*(*x*) to each take one of several functional forms (figure 2)
4.3
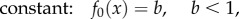

4.4
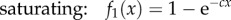

4.5
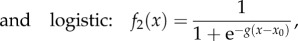
where *b*, *c*, *g* and *x*_0_ are arbitrary scaling constants. Hence, the net outcome of the above succession of functions (equation (4.1)) is a single flexible function that relates the parasite dose establishing in a newly infected recipient host (*P*), to the parasite dose within an infectious donor host (*L*), taking into account a range of potential nonlinearities at each step of the process. It is this composite function that we refer to as the ‘overall transmission function’.

**Figure 3.**
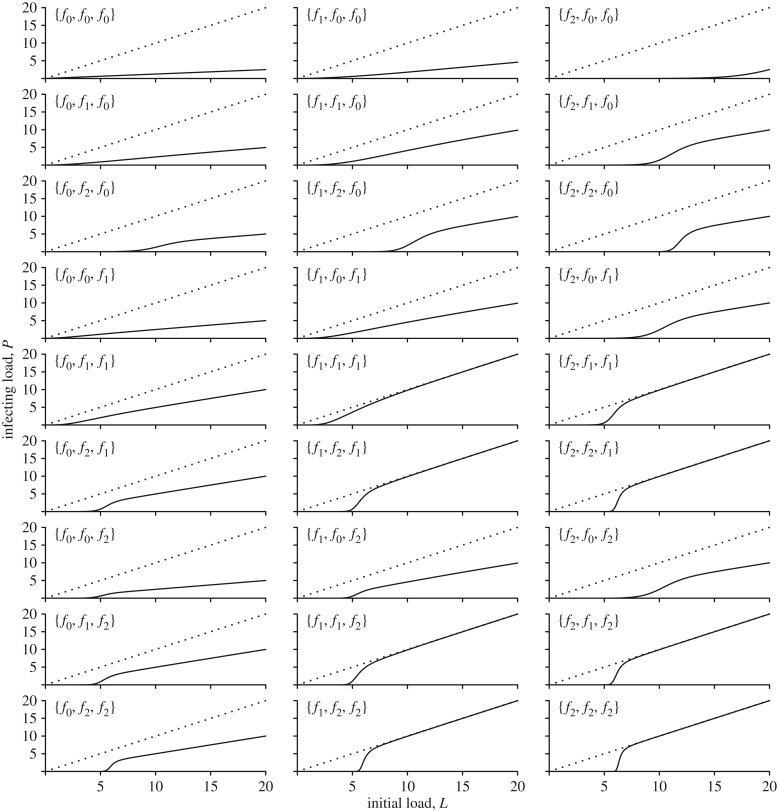
Overall transmission functions (relationship between initial infectious load in the donor host, *L*, and resultant infecting load in the recipient host, *P*) under all possible combinations of constant, saturating or logistic transmission functions, acting at each of the three stages in the transmission process described by equation (4.1). Each panel is marked with a label of the form {*p, d, r*}, which indicates the form of the transmission function (equations (4.3)–(4.5)) acting at the corresponding stage (*p*, *d* or *r*) of the overall transmission function in equation (4.1). The dotted lines show the 1 : 1 relationship. Parameter *b* = 0.5, *c* = 0.5, *g* = 2 and *x*_0_ = 5.

Overall, there are two main functional forms of the transmission function that emerge: approximately linear or rapidly accelerating to linear beyond a threshold load (figure 3). Linear, or near-linear, transmission functions arise for any combination of constant (*f*_0_) or saturating (*f*_1_) functions, regardless of the order in which they occur. However, if one of the stages includes a logistic process (*f*_2_) then this generates threshold-like behaviour in the overall transmission function, regardless of at which stage it acts (figure 3). Indeed, the order in which the different components act has little qualitative impact on the overall transmission function, although the order can affect it quantitatively, thereby altering the overall magnitude of transmission, if not its functional form. For example, if there is a logistic component acting early in the transmission process (e.g. {*f*_0_, *f*_0_, *f*_2_} in figure 3) then the threshold for rapid increase in transmission occurs at a much lower initial parasite load than if the logistic component occurs later in the transmission process (e.g. {*f*_2_, *f*_0_, *f*_0_} in figure 3).

## 5. Empirical measurement and the deconstructed *β*

In addition to highlighting the importance of nonlinearities, deconstructing *β* has a further advantage. Each stage and transition in the new framework (figure 1) identifies a clear biological point where empirical measurement is possible or serves to highlight those points in the transmission pathway for which we still lack the capacity to obtain measures. Parasite load in the host (stage 1) is one of the most frequently measured metrics. Some parasites lend themselves well to being directly counted, for example, ectoparasites such as ticks that are visible to the naked eye [43]. Large, multicellular endoparasites can also be enumerated easily, but this tends to require destructive sampling, although a non-destructive estimate of macroparasite load is also possible using endoscopy [44] and this has recently been pioneered in wildlife [45]. Microparasites, on the other hand typically require a sample from the host. Frequently, it is the parasite load of easily collected samples, such as blood, urine and saliva that is quantified, although this is not without inaccuracies; parasites are not distributed systematically or uniformly through the body of the host and are highly variable over time [46].

Measuring infective stage production (stage 2) for many macroparasites is straightforward: for example, using faecal egg counts such as McMaster or Kato-Katz [47]. For microparasites, capturing the instantaneous production of particles emitted can be more difficult, but it has been done successfully by quantifying influenza virus in human coughs [48]. Similarly, bio-aerosol sampling can measure the number of infectious particles in environmental samples at a cruder spatial scale [49]. Innovations in empirical measurements of the number of parasite infective stages produced have arisen from studies of ‘super-shedders’, those that produce many more infective stages than the average host, where measuring colony-forming units of bacteria have been used to quantify the heterogeneity in infective stages of *E. coli* produced in faecal pats [50].

Throughout the transmission process, whether the parasite is within the host or in the environment, similar methods are used for quantifying infective particles. Real-time quantitative PCR (qRT-PCR, or qPCR) is frequently used to measure the presence and concentration of the parasite DNA sequence and, in combination with tracking technology, such as proximity loggers, can determine when a contact leads to transmission [51]. One challenge, especially for viruses, is achieving adequate sensitivity for detection of the handful of infectious viral particles (as low as 1–10) that can be sufficient to create an infectious dose. Providing promise, however, to overcome such sensitivity issues is the use of digital PCR offering the potential to detect a single virus [52], although detection of viral nucleic acid alone is not proof of an active infectious virus, giving rise to erroneous transmission patterns when RNA fragments are ‘transmitted’ [53].

In many cases, the methods described above can be used to quantify the infective stages and the surviving parasite in the environment (stage 3), albeit with slightly different timescales of sampling. If the parasite follows an environmental transmission route (i.e. transition 2), is ingested via trophic transmission, or is vector-borne, then quantifying the dose acquired *could* be quite straightforward. In these cases, a simple count of the infective stages can be made in relation to food intake or vector biting rate [43]. The ease and accuracy of this empirical measurement is, however, parasite-specific. Measurement of environmentally transmitted helminths, for example, would require extensive sampling of the environment for larvae [54], without which the spatio-temporal aggregation typical of these infective stages could lead to values that over- or under-represent the surviving parasite.

Measuring the number of parasites contacting the host (stage 4) is one of the most challenging phases to measure empirically because, at a population level, a combination of surviving parasite load and host–parasite contact rate is required. New technologies have been used to quantify contact rates between individuals, for example, exploiting GPS technology in mobile phones to determine contact patterns in humans [55] and use of proximity loggers in wildlife [56], although quantifying the dose acquired by the recipient host (stage 5) remains an empirical challenge. Some host–parasite systems can, however, be used in this context: in a recent study Aiello *et al*. [51] undertook experiments with desert tortoises (*Gopherus agassizii*) and showed transmission likelihood was a function of time an infected and susceptible host spent together (usually in a burrow) and were able to estimate transmission patterns from data collected from proximity loggers. Notably the teleost-gyrodactylid systems where the metazoan parasites behave as if they were microparasites, can also be used in this context, where stage 5, the dose acquired, can be observed *in vivo* under a dissecting microscope using anaesthesia to immobilize the host [57,58]. Indeed this latter host–parasite system is ideal for monitoring the whole transmission process, where transmission is monitored as contacts between fish, and the parasite load (stage 1) and infective stages (stages 2–3) can be ascertained in the same manner as the acquired dose (see [58]).

An exciting possibility for tracking the entire transmission process in multiple host–parasite systems is the use of reporter parasites, where parasite numbers are correlated with light output to give a real-time and *in vivo* report of parasite load and location within the donor host [59,60]. Green fluorescent protein has been used previously as an *in vivo* measure of parasite load and dynamics [61], although with the disadvantage that the parasite in question will emit light even when no longer viable [62]. As such, a superior choice may be use of a self-bioluminescent reporter bacteria. These engineered bacteria only emit light when they are metabolically active and thus alive. Systems such as these can be used to measure parasite load *in vivo*, in real time, and to determine tissue distribution [63,64]. Hence, transmission can effectively be ‘seen’ in real time, allowing the whole transmission process to be quantified from initial parasite load (stage 1) through to recipient host's parasite load (stage 5), even allowing for monitoring of multiple parasites at once [65]. Bioluminescence imaging can overcome the issues of not knowing which tissue the parasite is in, provides a quantitative measure of parasite load and can also be measured in excreta, for example, faeces [64]. While this laboratory approach offers a method to quantify infective particles across all components defined in ‘deconstructed beta’, it is limited to laboratory host–parasite systems because such parasites are genetically modified and therefore need to be strictly contained. Clearly, deconstructing *β* can improve our ability to accurately measure each component of the transmission process, and for some host–parasite systems this will enable specific measurements at all stages. For others though, it allows us to identify those steps where measurement cannot be made and hence where estimation is needed. Understanding the exact point where further estimation is necessary can help to define clearer limits upon current estimates, leading to more accurate overall assessment.

## 6. Conclusion

Parasite transmission is a multi-faceted process [5]. Standard mathematical functions of transmission typically subsume most of this complexity into a simple linear term, with most of the biology of the interaction between an infectious agent and susceptible recipient host being captured by a single, composite parameter, *β*. While these approaches have been vital for developing a broad understanding of host–parasite dynamics, in particular allowing the development of analytically tractable general theory, there are many potentially important aspects of transmission biology that are inevitably ignored by these standard approaches. Here, we have sought to deconstruct the transmission process, breaking it down into its fundamental components, to develop a generalized framework applicable to any parasite-transmission scenario, which explicitly separates out key stages and transitions at which different sources of nonlinearity may act. Here, we show how each stage may be influenced, to different extents, by the combination of donor and recipient host factors, aspects of the parasite itself, and of the environment in which transmission occurs. Such details of all these aspects are inevitably overlooked by standard transmission theory. Furthermore, by exploring mathematically the potential implications of these various nonlinearities for the functional form of the overall transmission function, we show how threshold-like behaviours in transmission can easily emerge, potentially driving aspects of infection dynamics at both the individual and population levels that would otherwise be overlooked by traditional, linear transmission models.

The functional form of the nonlinearities at each transition in the transmission process is clearly important in determining the relationship between parasite burdens in the infectious donor host and recipient infected host. In particular, if one of the steps follows a logistic (sigmoidal) form, then that function dominates the transmission behaviour, driving highly nonlinear, threshold-like behaviours in transmission. Under these conditions, low parasite loads in the infecting donor host could generate very low infection loads in the recipient host. It may be that these low-level infections are sufficient to maintain transmission, but do so undetected (e.g. due to low infection titres), giving rise to chronic, ‘covert’ infections that circulate in the absence of obvious clinical signs [66,67]. However, if infection load is able to reach sufficiently high levels in a given host, the threshold-like behaviour that emerges from a logistic-shaped component of transmission could drive the occurrence of relative ‘super-shedder’ individuals, and potentially drive rapid (re-)emergence of infection at the population scale. Importantly, such nonlinearities, and their consequences at the individual and population level would be overlooked by the conventional, linear frameworks of transmission. Therefore, we suggest that explicit consideration of the functional form of these transmission processes is vital for understanding where the key nonlinearities lie, and therefore the likely effects of differently targeted control measures (e.g. should control be applied to reduce infectious dose in infected individuals, to block parasite production, to alter host–host contact or reduce susceptibility of potential recipient hosts through prophylactic treatment?).

Here, we have sought to deconstruct transmission into a series of fundamental stages and transitions representative of all transmission scenarios. Inevitably, though, there are other areas in which additional complexities can arise. In particular, there is potential for heterogeneities to arise in other aspects of the transmission process that can alter overall transmission dynamics. For example, the transmission process described above assumes that an infectious individual is only infected with one parasite genotype, whereas in reality, infected hosts will often have a community of parasites of different genotypes and phenotypes. In the simplest case, it would be possible to apply and parameterize this framework for each genotype separately, although that would assume there is no interaction between them. In reality, there would likely be either direct competition among genotypes and/or immune-mediated interactions, so more realistic transmission models would have to take this into account.

In addition to heterogeneities among the parasite population, there may also be considerable heterogeneities among both donor and recipient hosts. Infectious donors have differing infection levels due to a variety of reasons, including their own original degree of parasite susceptibility, the parasite infective dose they acquired, and subsequent within-host–parasite replication or immune response-mediated parasite losses. For macroparasites (e.g. helminths), such variation in intensity of infection in host populations is often best represented empirically by a negative binomial distribution [68,69], and similar distributions may apply for microparasites, for example *Staphylococcus aureus* [70], but other distributions are possible. Similarly, variability among susceptible recipient hosts is likely to be important in shaping transmission dynamics. For a given acquired dose, therefore, there are likely to be different resultant established parasite loads in different recipient hosts, depending on their condition, immune status, genetics and previous exposure to the parasite or other infections. It is important to note that this established dose refers only to those parasites establishing from this single infection event not to the recipient's entire parasite load. The newly infected recipient host does not simply become a new donor host (no arrow links stage 5 back to stage 1). To complete the loop, we would need to take account of the swathe of within-host processes and states described above.

Finally, we have ignored explicit consideration of heterogeneities or nonlinearities arising at the contact phase of transmission (stage 4), as this is the one stage that has received most attention to date. The fundamental difference between the two standard formulations of transmission (density-dependent and frequency-dependent) arises through different assumptions about how contact rates scale with host density [4,6]. Furthermore, various approaches have previously been used to account more explicitly, to a greater or lesser extent, for heterogeneities in contact, for example by using individual-to-individual contact networks [71,72], or ‘Who Acquires Infection From Whom’ matrices, for example in the transmission of HIV [23]. Although outside the scope of this paper, it would be informative to develop agent-based models that incorporate such heterogeneities in parasite, host and contact rates, to explore their importance in driving host–parasite dynamics at the population scale, the consequences for control, and the extent to which those results differ from those predicted by the standard formulations.

The current, standard formulations of transmission, the use of *β* as a composite transmission parameter, and the assumptions of linearity in transmission that it implies, will rightly continue to be used, not least because these approaches are mathematically highly convenient, and will continue to be important in developing our understanding of infection transmission and control. However, there are clear limitations with those approaches, glossing over potentially important aspects of transmission biology that could underlie, for example, the occurrence of extreme super-spreading events, or the ‘stubbornness’ of lingering cases often seen towards the end of control campaigns (for example, in the case of the on-going global polio eradication initiative). Considering in more detail the various stages that these transmission functions represent, and the potential factors that could drive nonlinearities and heterogeneities in those processes, may provide a more nuanced view of transmission, thereby enabling a more complete understanding of parasite dynamics and transmission in the future.
